# Malacological Survey and Spatial Distribution of Intermediate Host Snails in Schistosomiasis Endemic Districts of Rwanda

**DOI:** 10.3390/tropicalmed8060295

**Published:** 2023-05-28

**Authors:** Joseph Kagabo, Chester Kalinda, Project Nshimiyimana, Jean Bosco Mbonigaba, Eugene Ruberanziza, Elias Nyandwi, Nadine Rujeni

**Affiliations:** 1 School of Health Sciences, University of Rwanda College of Medicine and Health Sciences, KG 11 Ave., Gasabo, Kigali P.O. Box 3286, Rwanda; 2 Bill and Joyce Cummings Institute of Global Health, University of Global Health Equity (UGHE)|Kigali Heights, Plot 772 KG 7 Ave., Kigali P.O. Box 6955, Rwanda; ckalinda@ughe.org; 3 Rwanda Biomedical Centre (RBC) KG 644 St., Kigali P.O. Box 7162, Rwanda; 4 The End Fund, 2 Park Avenue, 28th Floor, New York, NY 10016, USA; 5 Centre for Geographic Information Systems and Remote Sensing (CGIS), University of Rwanda College of Science and Technology, KN 67 Street, Nyarugenge, Kigali P.O. Box 3900, Rwanda

**Keywords:** *Biomphalaria*, *Bulinus*, malacology, cercariae, schistosomiasis

## Abstract

Background: Schistosomiasis, due to *S. mansoni*, is prevalent in Rwanda. However, there is a paucity of information related to the abundance, species, distribution, and infectivity of *Schistosoma* intermediate host snails. Methods: Snails were collected from 71 sites, including lakeshores and wetlands. Snails obtained were morphologically identified, and cercariae were shed using standard procedures. Cercariae were molecularly characterized using PCR. GPS coordinates were used to generate geospatial maps of snail distribution that were overlaid with geospatial distribution of schistosomiasis among pre-school children in the same areas. Results: Overall, 3653 snails were morphologically classified as *Bulinus* spp. and 1449 as *Biomphalaria* spp. A total of 306 snails shed cercariae, 130 of which were confirmed as *S. mansoni* cercaria by PCR. There was no significant difference in the proportion of *S. mansoni* cercariae in wetlands compared to lakeshores. Conclusion: Rwandan water bodies harbor an important number of snails that shed *S. mansoni* cercariae. Furthermore, a strong spatial correlation was observed between the distribution of schistosomiasis in children and the spatial distribution of snail infectivity with *S. mansoni*. The presence of *Bulinus* spp. Suggests a potential risk of *S. haematobium*, although molecular analysis did not show any current transmission of this parasite.

## 1. Introduction

Human schistosomiasis, a snail-transmitted parasitic infection caused by blood flukes (also known as schistosomes) in the genus *Schistosoma*, remains a major public health problem in the tropical regions of the world. Though the majority of the world’s great neglected tropical diseases (NTDs) have decreased sharply in prevalence in recent years [1], the opposite is the case for schistosomiasis, with an estimated 258 million cases [2], which is over 25% higher than estimated 50 years ago. Furthermore, the impact of schistosomiasis as estimated by disability-adjusted life years (DALYs) has been re-appraised and found to be 4–30 folds higher than suggested by earlier estimates [3,4]. Of the world’s estimated 258 million cases, 85% occur in sub-Saharan Africa [2]. It is estimated that 500–600 million more people are at risk of contracting the infection [5]. Among the schistosome species that cause human schistosomiasis, four, namely, *Schistosoma* *mansoni*, *S. intercalatum*, *S. mekongi*, and *S. japonicum*, are responsible for causing intestinal schistosomiasis, while *S. haematobium* causes urogenital schistosomiasis. The three major schistosome species, namely *S. mansoni*, *S. haematobium*, and *S. japonicum*, are responsible for most of schistosomiasis cases worldwide [6]. However, *S. mansoni* is probably the most widespread and the most important from a public health perspective [5,7]. *S. mansoni* is found in many countries in Africa, in South America (Brazil, Surinam, and Venezuela), in the Caribbean (including Puerto Rico, St Lucia, Guadeloupe, Martinique, Dominican Republic, Antigua, and Montserrat), and in parts of the Middle East.

In sub-Saharan Africa, *S. mansoni* infects nearly 100 million people, mostly children, and results in up to 70 million disability-adjusted life years (DALYs) lost annually, exceeding those lost due to malaria or tuberculosis and almost reaching the DALYs lost from HIV/AIDS [7,8].

The symptoms of this disease are caused by the body’s reaction to the eggs produced by the worms, not the worms themselves, and the parasite eggs that become trapped in the body tissue cause an immune reaction [9]. Intestinal schistosomiasis is a debilitating disease often characterized by abdominal pain and discomfort, diarrhea and sometimes blood in the stool, and, in severe cases, hepatic portal hypertension [9]. In children, it may result in impaired physical growth and cognitive ability and a drop in physical performance [3,10]. Other effects of the disease include fibrotic responses to schistosome eggs trapped in the intestines, liver, and other organs of the infected person [11] and malnutrition, especially in children, which may retard physical growth [12]. Liver enlargement is common in advanced cases and is frequently associated with accumulation of fluid in the peritoneal cavity and hypertension of the abdominal blood vessels, and in such cases, there may also be enlargement of the spleen [9]. The classic sign of urogenital schistosomiasis is hematuria (blood in urine). Fibrosis of the bladder and ureter and kidney damage are sometimes diagnosed in advanced cases. Bladder cancer is another possible complication in the later stages of urogenital schistosomiasis. In women, urogenital schistosomiasis may present with genital lesions, vaginal bleeding, pain during sexual intercourse, and nodules in the vulva. In men, urogenital schistosomiasis can induce pathology of the seminal vesicles, prostate, and other organs. Urogenital schistosomiasis may also have other long-term irreversible consequences, including infertility [9]. Rarely, the parasite eggs can travel to the brain and cause seizures, paralysis, and spinal cord inflammation [13].

Human schistosomiasis is transmitted through freshwater or amphibious gastropod snails, and, in the case of *S. mansoni*, the freshwater pulmonate snails in the family Planorbidae and the genus *Biomphalaria* are responsible for its transmission [14].

Several species of *Biomphalaria* are known to transmit the parasite in Africa, but the most prominent among them is *B. pfeifferi*, a widespread inhabitant of streams and other small water bodies across sub-Saharan Africa [14].

In Rwanda, early malacological studies identified the freshwater snails *Biomphalaria* spp. as the intermediate hosts involved in the transmission of *S. mansoni* in Rwanda [15]. This was in tandem with the study results of 2012 from the Eastern provinces of the country, which showed high numbers of *Biomphalaria* spp. infected with *Schistosoma* cercariae [15].However there is a lack of tangible information from the studies conducted in Rwanda about the specific species involved in the transmission of *S. mansoni* in Rwanda, even though one report indicated *B. pfeifferi* as the main species transmitting the parasite around Lake Ruhondo [16]. It is observed that compatibility of local snail populations with schistosomes is a key determinant in schistosomiasis transmission success. The greater the compatibility, the more successfully the parasite will establish in the snail host and produce cercariae, which are then released into the aquatic environment for transmission. The lifecycle of schistosomes involves the release of parasite eggs in human urine or feces, the hatching of the eggs in water, the development of miracidia that infect snails, the production of cercariae that leave the snail and penetrate human skin, and the migration of the adult worms to the veins of the bladder or intestine [17].

Schistosomiasis control programs in several sub-Saharan African countries have mostly relied on preventive chemotherapy or mass administration of praziquantel to humans, mostly primary school-aged children [18,19,20]. However, studies have shown the inability of praziquantel to attain a 100% curative rate with both single and multidose regimens [21,22]. Furthermore, in endemic areas, re-infection occurs rapidly since communities rely on contaminated waters for their household or professional activities [23]. The WHO has set targets to eliminate schistosomiasis as a public health disease by 2030 [24]. This is only possible if countries adopt a multi-pronged approach, combining treatment with snail control, health education, Water-Sanitation and Hygiene (WASH) improvement, and multi-sectoral collaboration [25]. Therefore, the collection of robust data related to infection transmission sites and hotspots based on the distribution of intermediate snail hosts remains vital in accelerating progress towards attainment of control and elimination goals. Earlier modelling studies have shown schistosomiasis transmission sites and infection hotspots in Rwanda [26,27]. Furthermore, nationwide schistosomiasis surveys of *Schistosoma mansoni* infection conducted in 2020 showed the disease endemic areas in parts of the country [28]. On the other hand, studies conducted elsewhere have suggested that transmission of schistosomiasis is significantly dependent on the distribution of snail intermediate hosts [29,30].

Further recent studies conducted have focused on assessing the prevalence and intensity of *S. mansoni* infections in various populations, mostly among the pre-school children in Rwanda, and found high prevalence and intensity of *S. mansoni* in Rwanda [16]. Another study conducted in Bugesera District in Eastern Province among school-aged children also showed a high prevalence of *S. mansoni* infections, with a mean infection intensity of 81.4 eggs per gram of feces [31], an indication that *S. mansoni* is prevalent in Rwanda.

To control the spread of schistosomiasis in Rwanda, various initiatives have been implemented. One such initiative is the Rwanda National Schistosomiasis Control Program, which was established in 2011 with support from the World Health Organization (WHO). The program aims to reduce the burden of schistosomiasis in Rwanda by implementing mass drug administration (MDA) campaigns in high-risk areas, improving access to safe water and sanitation facilities, and promoting behavior change through health education campaigns.

In addition, other initiatives have focused on improving the surveillance and monitoring of schistosomiasis in Rwanda. For example, the Rwanda Biomedical Centre (RBC) has established a national schistosomiasis database to collect and manage data on the prevalence and distribution of the disease in the country. The database is used to inform the development and implementation of control strategies, as well as to evaluate the effectiveness of interventions.

Overall, while significant progress has been made in controlling the spread of schistosomiasis in Rwanda, ongoing efforts are needed to reduce the burden of this disease on affected communities. These efforts should include a continued focus on MDA campaigns, as well as broader interventions to improve access to safe water and sanitation facilities and to promote behavior change through health education campaigns.

However, snail control programs cannot be effectively implemented in the absence of robust information on their distribution and infectivity. This study sought to understand the distribution of snail intermediate hosts of *Schistosoma* in Rwanda, their infectivity, and the potential for schistosomiasis transmission to better inform the national control program.

## 2. Materials and Methods

### 2.1. Study Area

The study was conducted in 15 schistosomiasis-endemic districts of Rwanda. The study was done between July and December 2019, and 71 sites were selected based on the nation-wide prevalence mapping for schistosomiasis [32] and incidence data. The layer of endemic areas was intersected with layers of lakes, multipurpose water dams, fishponds, and/or important wetlands (wetland of ≥0.7 ha hosting a socioeconomic activity, mostly irrigated agriculture) to define sampling sites.

### 2.2. Snail Sampling and Cercariae Shedding

Sampling was carried out by trained technicians using a 0.2 mm standard scoop net with a 3-m-long metallic handle, as described by Madsen et al. [33], or occasionally using gloved hands and plastic spoons for effectiveness and precision, as previously described by Ofoezie [34]. At each sampling site (I), the sampling plot was designed as rectangular, measuring 10 m × 20 m (200 m^2^) in the wetlands and rice fields and 10 m along lakeshores. At each site, snails were collected for only 30 min. Sampled snails collected from each of the sites were placed in separate plastic containers and appropriately labeled and transported to the nearby field laboratory, where morphological identification was done according to Brown [14]. Furthermore, each sampling location was georeferenced using a handheld Garmin Etrex Receiver GPS machine. The shedding of snails was done by exposing snails placed individually in a culture plate containing 5 mL of distilled water to sunlight for 20 min according to the methods suggested by Gumble et al. [35]. Thereafter, the wells of the culture plates were examined for the presence of cercariae under a light microscope at 300× magnification [36]. Snails that did not shed cercariae after exposure were re-exposed to the sunlight on the second day, then those that never shed cercariae were crushed and checked for developing cercariae [37]. The shed cercariae were later stored in a solution of 70% alcohol before molecular characterization.

### 2.3. Molecular Characterization of Cercariae

A total of 306 cercaria specimens, suspected to be of *Schistosoma* spp. based on their morphological characteristics, were further analyzed at the molecular level. Briefly, DNA was extracted from each sample using DNeasy Blood & Tissue Kit (QIAGEN, Hilden, Germany). The extracted DNA from each of the 306 samples was subjected to amplification of species-specific DNA by PCR using the primers SM-F/R for detecting *S. mansoni* and primers ShF and CRI for detecting *S. haematobium*.

For detecting *S. mansoni*, PCR was carried out using forward primer SM-F (5′-GAGATCAAGTGTGACAGTTTGC-3′) and reverse primer SM-R (ACAGTGCGCGCGTCGTAAGC). It amplified 350 bp fragments from the tandem repeated DNA sequence of *Schistosoma* *mansoni* [38]. For the detection of *S. haematobium*, PCR was carried out using two sets of primers; the first set of primers was constituted of the forward primer ShF (5′-AGTCGTGTCGATTTTAA-GAC-3′) and the reverse primer CR (CCAACCATAAACATATGATG); it amplified 365 bp sequences of the cytochrome c oxidase subunit 1 (cox1) gene of *S. haematobium* [39]. The second set of primers comprised the forward primer Dra1F (GATCTCACCTATCAGAC-GAAAC) and its reverse primer Dra1R (TCACAACGATACGACCAAC), and it amplified 121 bp of a highly repeated sequence of *S. haematobium* [40]. The PCR was carried out in a final volume of 20 µL with 10.7 µL nuclease-free water, 4 µL Flex buffer, 2 µL of MgCl2, 0.4 µL of dNTPs, 0.4 µL of each primer (forward and reverse), 0.1 µL of GoTaq enzyme, and 2 µL of DNA template. The reaction was performed initially at 95 °C for 3 min followed by 35 cycles, each consisting of 94 °C for 30 s, 60 °C for 30 s, 72 °C for 45 s, and a final cycle of 72 °C for 8 min. The PCR products were electrophoresed in 2% agarose in 1x TAE buffer and 1/10,000 of SYBR Safe DNA stain and then visualized in a UV transilluminator (NuGenius by SYNGENE). On the gel electrophoresis, *S. mansoni* was confirmed when bands were obtained exactly at 350 bp using the GeneRuler 100 bp DNA Ladder (Thermo-scientific). For all the analyses performed in all the samples using DNA-amplified species-specific primers for *S. haematobium*, none of them gave a band corresponding to the 121 bp size of the highly repeated sequence of *S. haematobium* when using the primers Dra1F/Dra1R nor a band corresponding to the 365 bp size sequence of cox1 gene of *S. haematobium* using the primers ShF and CR. The results of this analysis confirm that all the cercariae being referred to under this sub-section are regarded as mammalian schistosome cercariae.

### 2.4. Human Infection Data Analysis

Data on the infection of pre-school-aged children (PreSAC) was collected from all the 15 districts where snail sampling had been conducted. The data from a parasitological study on PreSAC [41] were used to assess and determine the link between human infection and snail distribution and infectivity.

### 2.5. Parasitological Assessments

Urine samples were collected and tested for schistosomiasis circulating antigens using the Point-Of-Care Circulating Cathodic Antigen (POC-CCA or CCA), following the manufacturer’s instructions. CCA test results were recorded as negative (− or trace) or positive (+, ++, or +++ according to the intensity of the test line in comparison to a test control). Stool samples were also collected and tested using the Kato-Katz method for the detection of patient infection, following published protocols [28]. A single specimen was collected from which 2 slides were prepared and read by 2 laboratory technicians independently. Ten percent (10%) of all the slides were retested by the National Reference Laboratory senior technicians for quality control. The POC-CCA estimate was considered as the overall prevalence given the high sensitivity of the assay [32]. However, KK results were considered for infection intensity, where raw fecal worm egg count (FWEC) was multiplied by 24 to estimate the eggs per gram [42,43]. The level of infection intensity was determined based on WHO guidelines [7]. This previously published study [41] was compared with the molecular data results from cercariae and confirmed the presence of *S. mansoni* in the surveyed districts.

### 2.6. Data Analysis

Data analyses were executed using the Statistical Package for Social Sciences software version 21 (SPSS.21). Descriptive statistics were used to determine the abundance and distribution of snails in the different water bodies and geographical locations. Comparisons between water bodies was done using Chi-square analysis of proportions.

The geographical distribution of the snails was mapped by displaying geographical coordinates (X, Y data) using Esri GIS software known as ArcGIS 10.5. Those geographical coordinates were collected during fieldwork using the Garmin Etrex Receiver global positioning system (GPS) with an estimated accuracy of ±3 m [28].

## 3. Results

### 3.1. Snail Species Collected

**Figure 1:**
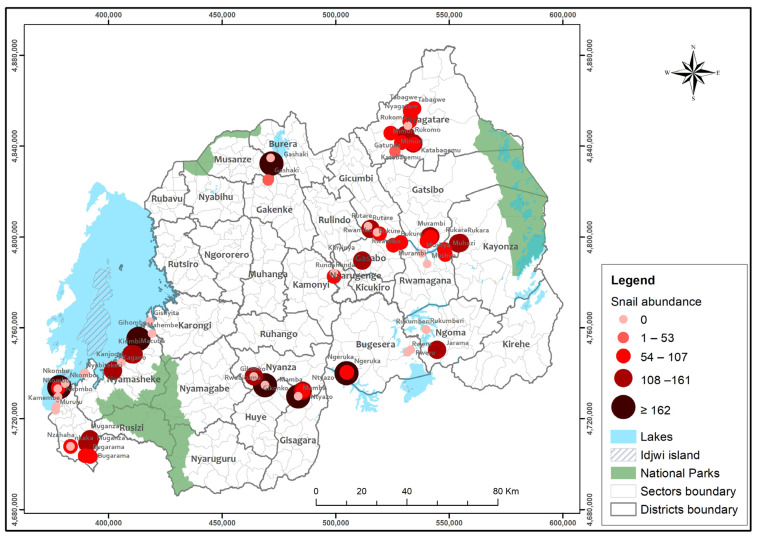
Snail Spatial distribution from 71 surveyed sites.

A total of 5102 freshwater snails were collected from 71 sites, 28 of which were along the lakeshores, while 43 were from the wetlands. Furthermore, of the collected snails, 3653 (71.60%) were classified as *Bulinus* spp. and 1449 (28.40%) as *Biomphalaria* spp. (Supplementary Table S1). In addition, 3914 (76.72%) were collected from wetlands, while 1188 (23.28%) were collected from lakeshores. The spatial distribution indicated that most of the sampled sites had snails, but the abundance differed between districts (Figure 1). Out of 71 sites sampled, 42 sites (59.2%) had snails, and 29 (40.8%) sites had no snails. Almost all the sampling sites had live snails, and many sites accounted for the two snail species of *Biomphalaria.* spp. and *Bulinus*. spp. (Supplementary Figure S1). This study also identified both *Biomphalaria* and *Bulinus* snail species during collection and screening, which were distinguished by their shell morphology and the taxonomic identification keys as described by [14] (Supplementary Figures S2a,b and S3a).

### 3.2. Cercariae Shedding Per Snail Species

Of the 435 *Biomphalaria* spp. snails examined for cercaria shedding, only 119 (27.4%) shed cercariae, while 489 (44.6%) of the 1096 *Bulinus* spp. snails shed cercariae. Split per waterbody, 32.8% of the shedding *Bulinus* spp. were found along lakeshores, while 48.2% were found in wetlands (Table 1). On the other hand, 42% versus 23% of shedding *Biomphalaria* snails were in lakeshores versus wetlands, respectively. However, no significant difference was found regarding the proportion of *S. mansoni*-positive cercariae between the waterbodies (47.4% versus 40.4%, X^2^ = 1.2, *p* = 0.274).

### 3.3. *Schistosoma* Cercaria Molecular Characterization

**Figure 2:**
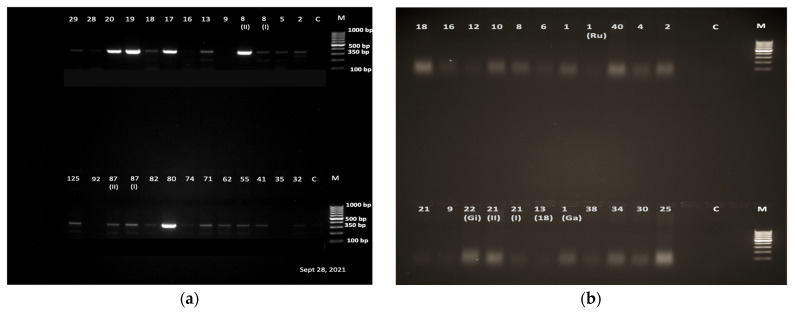
(**a**) Gel electrophoresis results of DNA products obtained using primers specific for *S. mansoni*. Lane M, DNA ladder 100 bp; Lane C, negative control; Lanes with digit numbers correspond to the samples; (**b**) Gel electrophoresis results of DNA products obtained using primers specific for *S. haematobium*. Lane M, DNA ladder 100 bp; Lane C, negative control; Lanes with digit numbers correspond to the samples.

Of the 306 samples analyzed with PCR, 130 were confirmed to be positive for *S. mansoni*, as evidenced by the molecular size of the PCR products on the gel (Figure 2a). The remaining 176 samples that tested negative for the presence of *S. mansoni* using the primers SM-F/R were amplified using ShF/CR and Dra1F/Dra1R primers for the detection of *S. haematobium*. However, none of them gave a band corresponding to the 121 bp of the highly repeated sequence of *S. haematobium* when using the primers Dra1F/Dra1R nor a band corresponding to the 365 bp sequence of cox1 gene of *S. haematobium* using the primers ShF and CR (Figure 2b).The results of this analysis therefore confirms that all the cercariae being referred to in this sub-section are regarded as mammalian schistosome cercariae.

### 3.4. *Biomphalaria* and S. mansoni Spatial Distribution

**Figure 3:**
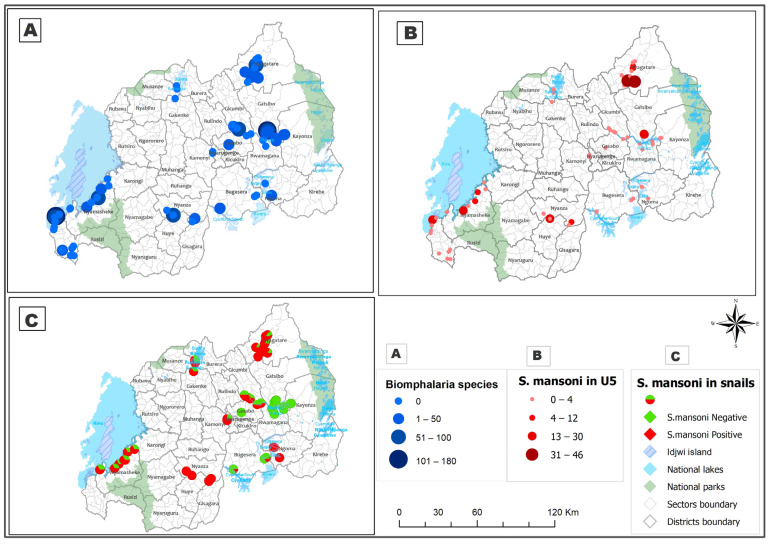
The spatial distribution of *S. mansoni*-shedding *Biomphalaria* snails and schistosomiasis distribution among pre-school-aged children (PreSAC).

The spatial distribution of *Biomphalaria* snails that shed *S. mansoni* coincided with the spatial distribution of *S. mansoni* infection among PreSAC, as shown on Figure 3.

This coincidence shows that there is a spatial correlation between the distribution of *Biomphalaria* snails that can shed the parasite *Schistosoma* *mansoni* and the distribution of *S. mansoni* infection among PreSAC (pre-school-aged children). This further suggests that the presence and density of these snails in certain areas may be a contributing factor to the transmission of *S. mansoni* to humans, particularly among young children [44]. Therefore, understanding the spatial distribution of these snails could potentially aid in the development of more targeted and effective strategies for controlling the spread of this parasitic infection.

## 4. Discussion

It is well understood that snails play a crucial role in the transmission of parasitic diseases, including schistosomiasis, in many parts of sub-Saharan Africa. In Rwanda, the prevalence of schistosomiasis is high, and snails are the main intermediate hosts for the parasites that cause this disease. Therefore, understanding the distribution and transmission potential of snails in water contact areas has been essential for controlling schistosomiasis in the country.

Several studies have been conducted in Rwanda to investigate the distribution and transmission potential of snails in water contact areas. For example, a study investigated the distribution and prevalence of snails in freshwater bodies in the eastern part of Rwanda [15].The study found that snails were present in 85% of the freshwater bodies surveyed, with *Bulinus globosus* and *Biomphalaria pfeifferi* being the most common species. The study also found that snail populations were influenced by factors such as water temperature, pH, and nutrient content. Another study by Ingabire et al. (2017) investigated the impact of human activities on snail populations and schistosomiasis transmission in a rural area of Rwanda. The study found that human activities such as agriculture, fishing, and domestic water use were important factors in the distribution and transmission of snails and schistosomiasis. The study recommended public health interventions such as mass drug administration and water and sanitation improvements to reduce the risk of infection.

The current study was designed to give insights about *Schistosoma* intermediate hosts (snails) distribution in waterbodies of Rwanda. This is the most comprehensive malacological assessment in the country to date. The results presented indicate the presence of both *Bulinus* and *Biomphalaria* intermediate host snails in various districts of the country. While further work is needed to confirm and correctly characterize the species of *Bulinus snails* available in Rwanda, our results indicate the presence and wide distribution of *Biomphalaria pfeiferi* and thus a risk of *Schistosoma mansoni*, as reported in earlier studies [26,27,41,45]. This work presented here follows the previously conducted nationwide parasitological surveys of 2007–2008, 2014, and 2020 that showed schistosomiasis infection prevalence per district ranging between 0 and 69.5% among school-aged children [28,45]. The results from the current study affirm the risks of *S. mansoni*. Furthermore, the presence of *Bulinus*. spp. may pose a potential risk of *S. haematobium* transmission.

Several earlier studies and predictive models focusing on the risk of schistosomiasis transmission have been based on human infections [26,27,41,45]. While control of schistosomiasis had previously been based on reducing morbidity among humans, understanding the distribution of snails becomes important in achieving the current disease elimination target. The current study shows that intermediate host snails are widely distributed in several wetlands and lakeshores of Rwanda. However, only a few sites showed the presence of *S. mansoni* in snails, and these sites should be targeted for snail control in addition to preventative chemotherapy to reduce the risks of disease transmission.

Although *Biomphalaria* snails co-occurred with *Bulinus* spp., our results show that there was no *Schistosoma* haematobium cercaria shed. The occurrence of *suspected Bulinus* spp. snails had earlier been reported in the northeastern part of Rwanda [45]. Given the possibility of confusing *Bulinus*. spp. with *Lymnaea*. spp., further molecular characterizations will confirm the identity of these snails and their impact on public health. Nevertheless, there is a potential for the transmission of *S. haematobium* in the case of parasite introduction in these areas due to human migration. The current study further observed that more snails were found in wetlands than along the shorelines, which was expected since wetlands host more stagnant waters and vegetations suitable for snails [18]. These wetlands, which include rice paddies, thus remain at high risk for schistosomiasis transmission. On the other hand, the observed number of snails on the shorelines may be due to the drifting of snails along the offshore lines, as observed in studies conducted in Tanzania and Zimbabwe along low lying waters [46,47].

Our mapping exercise indicated that waterbodies with snails that shed S. *mansoni* cercaria strongly correlate with the distribution of schistosomiasis among preSAC in Rwanda. The observation of snail populations along lakeshores and in wetlands thus suggests that an increase in human contact activities (such as farming) within these areas is likely to maintain schistosomiasis transmission and further expand transmission to new foci.

Studies done in Southern Africa [38,47] suggested that snails exposed to desiccation can survive the desiccation period and rebuild their populations to previously observed levels, suggesting that droughts between farming seasons (or rainy seasons) cannot interrupt schistosomiasis transmission along wetlands. Therefore, with the planned expansion of irrigation schemes to ensure food security [48,49], a close surveillance of schistosomiasis transmission is warranted, especially since predictive models of future schistosomiasis transmission show increased risk of infection in rice cultivation schemes [26]. Furthermore, landscapes with artificial and multipurpose water dams which are frequently visited by adjacent communities for agricultural purposes have also been identified as new foci or risk zones adding to traditional disease reservoirs [50].

In Rwanda, various water contact activities take place at lakeshores, such as bathing, fishing, washing clothes, and fetching water, whereas wetlands are mainly used for paddy rice farming. Overall, the *S. mansoni* shedding of snails observed in the current study in both wetlands and lakeshores tags schistosomiasis transmission sites and highlights the need for increased awareness to reduce the risks of infection. Furthermore, this study gives precisions that can be leveraged to introduce snail control interventions in our march towards schistosomiasis elimination as a public health problem in Rwanda. The study further considered the variability in Rwanda’s topography, with high mountainous areas in northern, southern, and western provinces, while the eastern area is relatively flat. As Rwanda moves towards schistosomiasis elimination, understanding the geographical distribution of intermediate host snails is key in planning and implementing strategies for schistosomiasis control. The mountainous nature of the environment in Rwanda suggested that snail distribution and abundance are likely to be fragmented into small, isolated populations. This is corroborated by a study conducted by Zhou [51], who suggested that fragmentation of the environment affects the spatial distribution of snails and thus reduces their population, as observed in the current study.

## 5. Conclusions

Results from this study suggest that both *Biomphalaria* and *Bulinus* snail species are abundant and widely distributed in Rwanda and that the contributing sources of local infections are lakeshores and wetlands, though the distribution of infected snails may not be the only target. Therefore, there is a need to investigate other external factors, for example, the temporal migration from other endemic areas/neighboring countries. It also appears that the existing snail control strategies in Rwanda are only restricted to smaller ecological zones where infections have been observed and confirmed in the recently conducted nationwide mapping surveys, which hinders the large-scale control efforts to eliminate the disease in Rwanda.

This study further highlights areas with the highest risks of *S. mansoni* transmission while confirming the absence of *S. haematobium* transmission. Understanding the abundance, distribution, and infectivity of snails is invaluable in our efforts to implement cost-effective schistosomiasis control interventions and surveillance. The study further recommends an assessment of the influence of environmental factors on the snail abundance and distribution in Rwanda to better tailor interventions.

## Figures and Tables

**Table 1:** Proportions of shedding snails per water body and per species.

Waterbody	*Bulinus* spp.		*Biomphalaria* spp.			
Lakeshore	Tested	Shed	Tested	Shed	PCR tested	PCR positive *S. mansoni*
	253	83 (32.8%)	103	43 (41.7%)	78	37 (47.4%)
Wetland	843	406(48.2%)	332	76 (22.9%)	228	92 (40.4%)

## Data Availability

The dataset leading to the writing of this manuscript is available upon request from the corresponding author.

## Supplementary Materials

The following supporting information can be downloaded at https://www.mdpi.com/article/10.3390/tropicalmed8060295/s1: Table S1. Snail abundance from the surveyed districts; Figure S1: Spatial distribution of snails by species from the endemic areas, colored by abundancies; Figures S2: (**a**,**b**) showing species cercariae types from the two snail species; Figure S3: The snail species morphological shapes as identified using standard keys.
