# Supplementary material for: Malacological Survey and Spatial Distribution of Intermediate Host Snails in Schistosomiasis Endemic Districts of Rwanda
Source: Trop Med Infect Dis. 2023 May 28;8(6):295. doi: 10.3390/tropicalmed8060295 (PMC10303441; doi:10.3390/tropicalmed8060295)

Figure S1: Spatial distribution of snails by species from the endemic areas, coloured by abundancies.

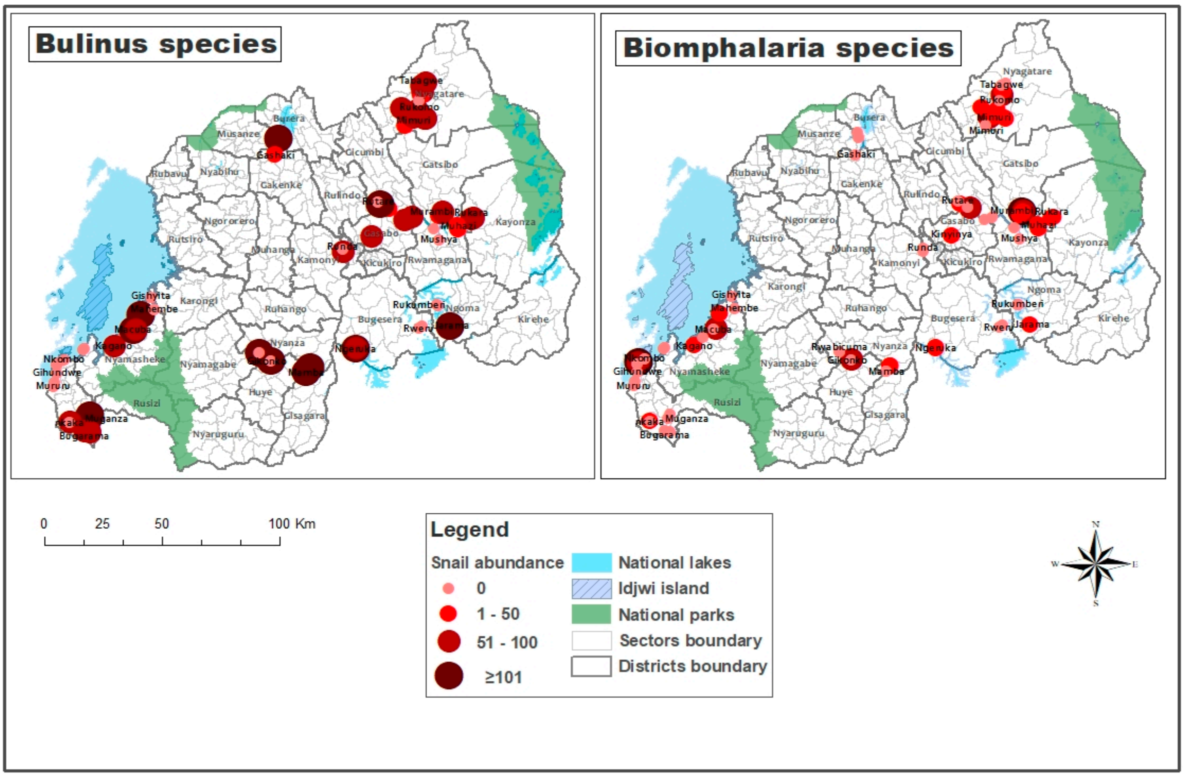

Supplement: Supplementary file 1 [file tropicalmed-08-00295-s001.zip › tropicalmed-2327010-Figure S1.pdf]
