# Supplementary material for: Malacological Survey and Spatial Distribution of Intermediate Host Snails in Schistosomiasis Endemic Districts of Rwanda
Source: Trop Med Infect Dis. 2023 May 28;8(6):295. doi: 10.3390/tropicalmed8060295 (PMC10303441; doi:10.3390/tropicalmed8060295)
Supplement: Supplementary file 1 [file tropicalmed-08-00295-s001.zip › tropicalmed-2327010-Figure S2.pdf]

Morphologically , snail species cercariae were observed under the microscope and were found to have differing characteristics.The Furcocercous cercaria from *Bulinus* spp and Gymnocephalous cercaria from Biomphalaria.spp(2)**.**

Their shapes are illustrated below- Figure S2.(a and b)

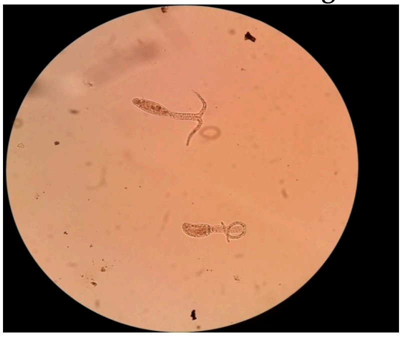

Figure S2.a showing the Furcocercous cercaria from *Bulinus* spp

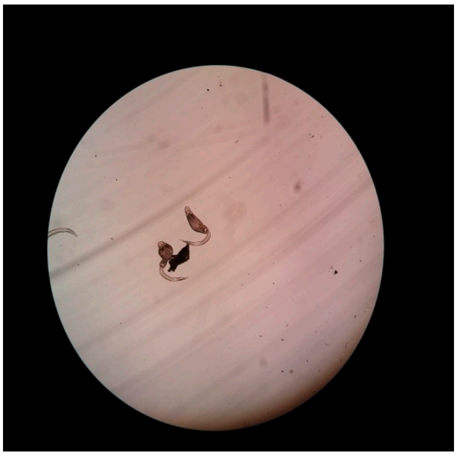

Figure S2.b showing the Gymnocephalous cercaria from *Biomphalaria* spp.
