# Supplementary material for: Malacological Survey and Spatial Distribution of Intermediate Host Snails in Schistosomiasis Endemic Districts of Rwanda
Source: Trop Med Infect Dis. 2023 May 28;8(6):295. doi: 10.3390/tropicalmed8060295 (PMC10303441; doi:10.3390/tropicalmed8060295)

Figure.S3.howing the B.*truncutus* **species that were collected along the wetland (rice paddies and along the lakeshores in Rwanda**

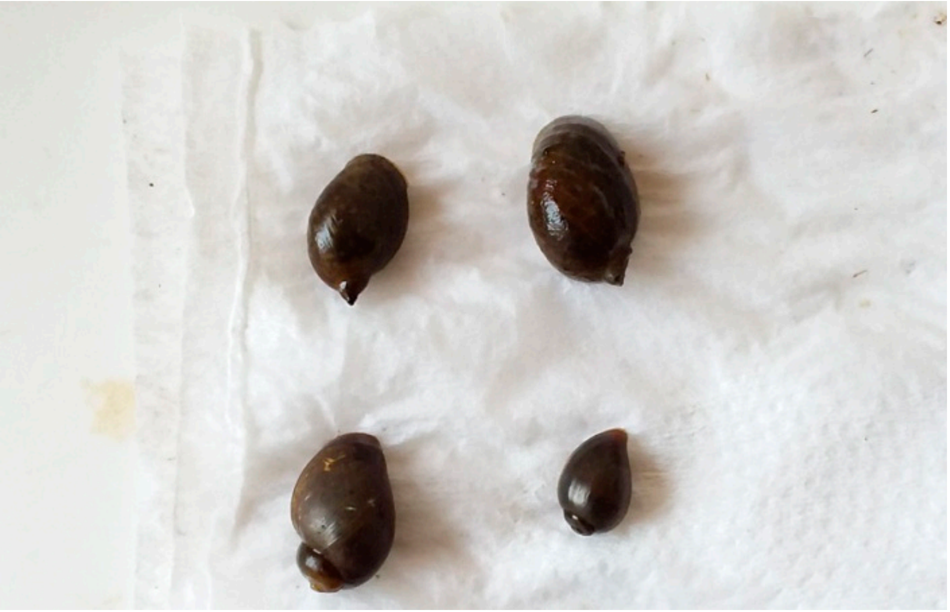

Supplement: Supplementary file 1 [file tropicalmed-08-00295-s001.zip › tropicalmed-2327010-Figure S3.pdf]
