# Supplementary material for: Malacological Survey and Spatial Distribution of Intermediate Host Snails in Schistosomiasis Endemic Districts of Rwanda
Source: Trop Med Infect Dis. 2023 May 28;8(6):295. doi: 10.3390/tropicalmed8060295 (PMC10303441; doi:10.3390/tropicalmed8060295)
Supplement: Supplementary file 1 [file tropicalmed-08-00295-s001.zip › tropicalmed-2327010-Table S1.pdf]

Table S1: Snail abundance from the surveyed districts

DISTRICT	BULINUS	BIOMPHALARIA	TOTAL
Bugesera	241	27	268
Gasabo	92	28	120
Gatsibo	158	248	406
Gicumbi	301	77	378
Gisagara	352	104	456
Kamonyi	91	0	91
Karongi	0	0	0
Kayonza	123	94	217
Musanze	257	0	257
Ngoma	123	17	140
Nyagatare	716	294	1010
Nyamasheke	364	179	543
Nyanza	271	46	317
Rusizi	486	293	779
Rwamagana	78	42	120
Total	3653	1449	5102
